# Targeting Anticancer Drug Delivery to Pancreatic Cancer Cells Using a Fucose-Bound Nanoparticle Approach

**DOI:** 10.1371/journal.pone.0039545

**Published:** 2012-07-11

**Authors:** Makoto Yoshida, Rishu Takimoto, Kazuyuki Murase, Yasushi Sato, Masahiro Hirakawa, Fumito Tamura, Tsutomu Sato, Satoshi Iyama, Takahiro Osuga, Koji Miyanishi, Kohichi Takada, Tsuyoshi Hayashi, Masayoshi Kobune, Junji Kato

**Affiliations:** 1 Fourth Department of Internal Medicine, Sapporo Medical University School of Medicine, Sapporo, Japan; 2 Division of Clinical Oncology, Sapporo Medical University Graduate School of Medicine, chuo-ku, Sapporo, Japan; 3 Division of Molecular Oncology, Sapporo Medical University Graduate School of Medicine, chuo-ku, Sapporo, Japan; City of Hope National Medical Center and Beckman Research Institute, United States of America

## Abstract

Owing to its aggressiveness and the lack of effective therapies, pancreatic ductal adenocarcinoma has a dismal prognosis. New strategies to improve treatment and survival are therefore urgently required. Numerous fucosylated antigens in sera serve as tumor markers for cancer detection and evaluation of treatment efficacy. Increased expression of fucosyltransferases has also been reported for pancreatic cancer. These enzymes accelerate malignant transformation through fucosylation of sialylated precursors, suggesting a crucial requirement for fucose by pancreatic cancer cells. With this in mind, we developed fucose-bound nanoparticles as vehicles for delivery of anticancer drugs specifically to cancer cells. L-fucose-bound liposomes containing Cy5.5 or Cisplatin were effectively delivered into CA19-9 expressing pancreatic cancer cells. Excess L-fucose decreased the efficiency of Cy5.5 introduction by L-fucose-bound liposomes, suggesting L-fucose-receptor-mediated delivery. Intravenously injected L-fucose-bound liposomes carrying Cisplatin were successfully delivered to pancreatic cancer cells, mediating efficient tumor growth inhibition as well as prolonging survival in mouse xenograft models. This modality represents a new strategy for pancreatic cancer cell-targeting therapy.

## Introduction

Pancreatic ductal adenocarcinoma is one of the most aggressive malignancies and has a dismal prognosis. It is estimated that pancreatic cancer mortality ranks eighth in cancer-related deaths worldwide. The overall 5-year survival rate of only 1% to 4% is due to the inability to detect this disease at an early stage, its aggressiveness, and the lack of effective conservative therapies [1]–[4]. Even those patients who are able to undergo surgical resection mostly relapse, resulting in a generally unfavorable outcome [3]. Although almost 80% of patients diagnosed at a highly advanced inoperable stage (IV) are treated with gemcitabine, gemcitabine in combination with Erlotinib, or FOLFIRINOX, the median survival time is reported to be only 5.7 months, 6.8 months, and 11.1 months, respectively [1], [5], [6].

One plausible explanation for the poor response of advanced pancreatic cancer is that systemic chemotherapy results in extremely inefficient delivery of anticancer drugs to the tumor because of its hypovascularity [7]. In an effort to overcome poor delivery of anti-cancer drugs, we have developed arterial infusion chemotherapy with gemcitabine and 5-fluorouracil for unresectable advanced pancreatic cancer after vascular supply redistribution via superselective embolization [8]. In a Phase I/II trial, an overall response rate of 33.3% and a median survival time of 22.7 months (95% CI; 9.5–24.5) was achieved, a better result than with intravenous gemcitabine monotherapy [1]. However, 2-year overall survival was still only 25% due to poor control of metastatic lesions [8]. This indicates that still more effective therapies are required. Specific delivery of anticancer drugs to cancer cells may result in improved efficacy.

Anti-cancer drug delivery specifically to cancer cells remains a major challenge. Several approaches, such as liposomes, polymers, polymersome, and micelles carrying anti-cancer drugs, have been utilized for the delivery of drugs to cancer cells, with the expectation of passive targeting through enhanced permeation and retention (EPR) effects [9]. However, lipid-based carriers have been reported to be rapidly cleared from the bloodstream by the reticuloendothelial system (RES) [9]. In order to overcome this issue, chemical modification of drug carriers with certain synthetic polymers has been frequently employed in an attempt to increase *in vivo* longevity [10]. The most popular and successful modification is coating with polyethylene glycol (PEG) to achieve “steric stabilization”, which hinders the interaction of blood components with their surface and reduces the binding of plasma proteins, toxicity, immunogenicity, and accumulation in the RES [11], [12]. One such example, is doxorubicin in PEG-coated liposomes (Doxil® and Caelyx®), which is widely used in clinical practice to treat solid tumors in patients with breast carcinoma [13]. However, recent evidence has shown that PEG, which was previously considered to be biologically inert, could still induce certain adverse effects through activation of the complement system [14]. Other approaches using polymer-based or organic nanoparticles (Abraxan®) are used clinically, but these are limited by the lack of controlled drug release at specific sites due to longevity in the blood stream, which leads to adverse effects [15].

Another way to actively target cancer cells is through the use of nanocarriers conjugated with molecules that bind to antigens or receptors on cancer cells [17], [18]; however, obstacles remain with this strategy, such as non-specific uptake by the RES and by non-targeted cells [9], [16]. For example, when antibodies are used in their native state for modification of nanocarriers, the Fc domain of an intact monoclonal antibody can also bind to the Fc receptors on normal cells, as occurs with macrophages, leading to increased immnunogenecity and uptake by the RES [19], [20]. Although the efficacy of these modification has been proven, lethal side effects have been also observed, likely due to non-specific binding [21] between the targeting agent and non-target moieties on the cell surface. Thus, a specific cancer cell-targeting carrier that does not undergo trapping by the RES or non-targeted sites is urgently required.

Accordingly, we focused on the biological characteristics of pancreatic cancer, especially fucosylated antigens, such as sialyl Lewis X-I (SLX) antigen and carbohydrate antigen-19-9 (CA19-9) which are found in the sera and tumor tissues of patients [22]–[25]. These are used as tumor markers for cancer detection and evaluation of treatment efficacy. Of several such fucosylated antigens, CA19-9 has been identified as a widely useful tumor marker for pancreatic adenocarcinoma because of its frequent elevation in this disease (∼80%) [26], [27]. It has also been shown that the postoperative survival rate is significantly worse in pancreatic adenocarcinoma patients whose CA19-9 levels are more markedly elevated [28].

Fucose, a deoxyhexose sugar, plays a physiological role in the modification of various molecules in mammals. For example, fucosylation plays an important role in blood group determination, immunological reactions, and signal transduction pathways [29]. Synthesis of fucose occurs via two major pathways [30]; i.e., *de novo* and salvage. In the former, GDP-fucose is synthesized from GDP-mannose by two enzymatic reactions. In the latter, free fucose derived from extracellular or lysosomal sources [31], or from dietary sources (or culture medium *in vitro*) is transported across the plasma membrane into the cytosol. Although the precise mechanisms remain unclear, increased levels of fucose are frequently found in the sera and urine of patients with cancer, including pancreatic cancer, colorectal cancer, and gastric cancer [32]–[34], which suggests that fucosylation is increased in cancer cells.

Enhanced expression of fucosyltransferases (FUTs) has also been reported in various cancers. For synthesis of CA19-9, FUTs add L-fucose in α(1,3) and α(1,4) linkage to sialylated precursors [35]–[37]. FUTs are key enzymes accelerating malignant transformation through the fucosylation of different sialylated precursors [32], [35]–[37]. It has been reported that enhanced activity of FUT3 is associated with increased metastatic potential of pancreatic adenocarcinoma cells [38], suggesting that fucosylation may play an important role in disease progression. These observations indicated a high requirement for L-fucose by various cancer cells [22], [24].

With this in mind, we have developed L-fucose-bound nanoparticles as vehicles for the delivery of anticancer drugs specifically to these cells *via* receptor-mediated endocytosis. We modified the size of the nanoparticles to allow penetration through the smallest capillary pores within the cancer vasculature, but not through the blood-brain barrier, via EPR effects [16]. Furthermore, in order to prevent non-specific trapping by the RES, hydrophilization of the liposome surface was carried out in an effort to prolong systemic retention and encapsulation of the anticancer drug, Cisplatin. Herein we report that intravenously injected L-fucose-bound liposomes containing Cisplatin can be successfully delivered to pancreatic cancer cells that express fucosylated antigens. This resulted in efficient tumor growth inhibition as well as prolonged survival in tumor-bearing mice.

## Results

### Production and Physicochemical Properties of L-fucose-bound Liposomes

**Figure 1:**
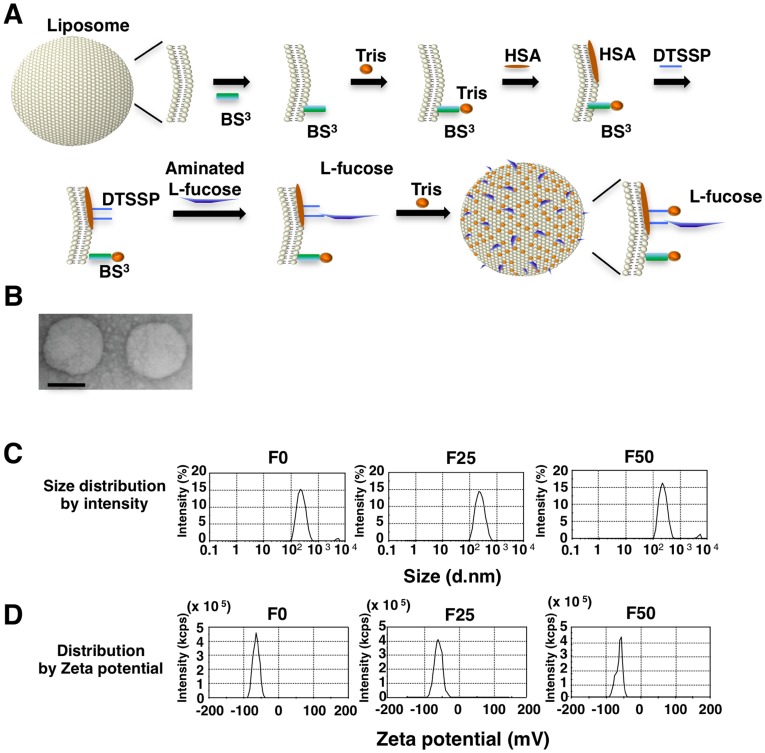
Production and physicochemical properties of L-fucose-bound liposomes. (**A)** Liposome preparation scheme showing sugar chains. HSA, BS^3^, Tris, and DTSSP denote the following, respectively: human serum albumin; bis(sulfosuccinimidyl) suberate; Tris(hydroxymethyl) aminomethane; 3,3-dithiobis (sulfosuccinimidylpropionate). (**B)** Electron microscopic image of L-fucose-bound liposome. Scale bar shows 50 nm. (**C, D)** Physicochemical characterization of Fuc-Liposome-Cy5.5. Average particle size **(C)** and zeta-potential **(D)** of liposomes that were prepared in water was determined by dynamic light scattering spectrophotometry.

Aminated L-fucose was cross-linked via 3,3′-dithiobis[sulfosuccinimidylpropionate] (DTSSP) to liposomes prepared by the modified cholate dialysis method to achieve a final concentration of 25 µg/ml (F25) or 50 µg/ml (F50). BS^3^ and Tris were then coupled on to hydrophilize the liposome surface (**Figure 1A**), which can prevent uptake by the RES in the liver and spleen and by macrophages and vascular endothelial cells, and can also prevent adsorption to opsonin proteins in plasma. Consequently, systemic retention of the liposomes is prolonged [39]. Examination by transmission electron microscopy showed that almost all L-fucose-bound liposomes (Fuc-Liposomes) were spherical in shape and, in the case of Cy5.5-encapsulation, were approximately 80 - 90 nm in size (**Figure 1B, C** **, and** **Table S1**). This particle size agrees well with measurements made by the Zetasizer Nano-S90 (**Figure 1C**). The zeta-potential, representing the negative electric charge of the liposome surface, was below -40 mV (**Figure 1D** **, and Table S1, S2**), which is sufficiently hydrophilized for stealth function. Particle size distribution remained stable after storage at 4°C for 6 months.

### Transfer of Fuc-Liposome-Cy5.5 and -FAM into Cancer Cells

**Figure 2:**
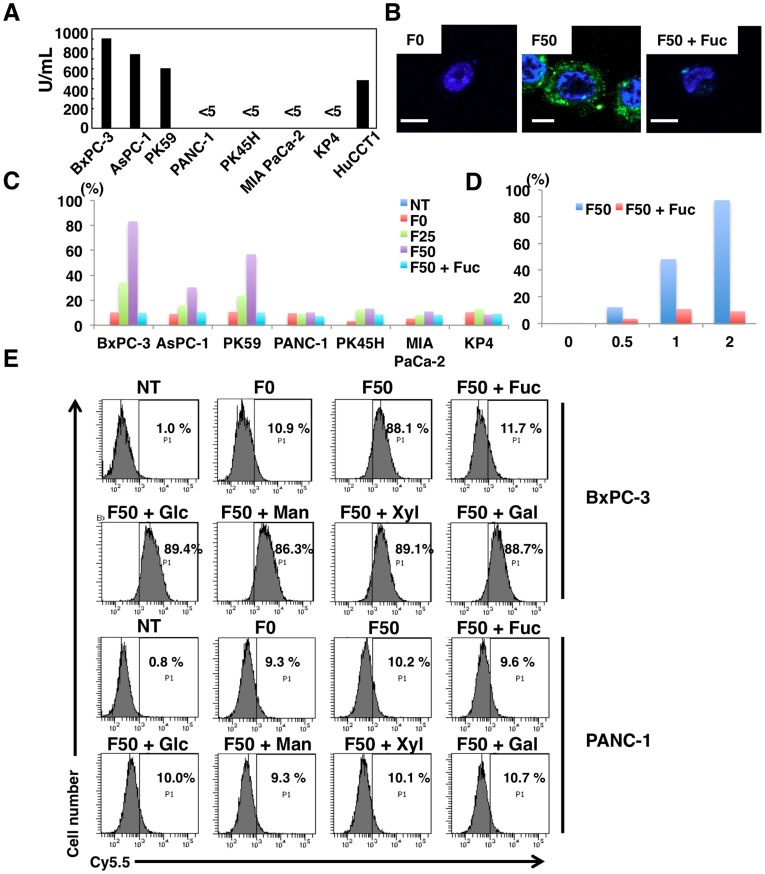
Transfer of Fuc-Liposome-Cy5.5 and -FAM into pancreatic cancer cells. (**A**) Concentration of CA19-9 secreted from different pancreatic cancer cell lines. Five million cells were incubated in serum-free medium for 48 hours and CA19-9 concentration was measured by ELISA. (**B)** BxPC-3 cells were incubated with Fuc-Liposome-FAM in the presence or absence of excess L-fucose for 2 hours, then washed and observed by confocal laser microscopy. Scale bar, 10 µm. (**C**) Flow cytometric analysis of Fuc-Liposome-Cy5.5-treated cells. BxPC-3, PK59, AsPC-1 (CA19-9 producing cancer cells) and PANC-1, PK45H, MIA PaCa-2, KP4 (CA19-9 non-producing pancreatic cancer cells) cells were treated with Fuc-Liposome-Cy5.5 for 2 hours with or without excess L-fucose and were analyzed by flow cytometry. Cy5.5 positive cells were indicated. NT, no treatment: F0, F0-Liposome-Cy5.5: F25, F25-Liposome-Cy5.5: F50, F50-Liposome-Cy5.5: F50+ Fuc, excess L-Fucose. (**D**) HuCCT1 (CA19-9 producing) cells were incubated with Fuc-Liposome-Cy5.5 for indicated hours in the presence or absence of excess L-fucose, then washed twice with phosphate-buffered saline and analyzed by flow cytometry. (**E**) Effect of monosaccharides on introduction of Cy5.5 into pancreatic cancer cells by Fuc-Liposomes. Flow cytometric analysis of Fuc-Liposome-Cy5.5-treated cells. Cells were treated with Fuc-Liposome-Cy5.5 or Lipsome-Cy5.5 for 2 hours with or without excess monosaccharides and were analyzed by flow cytometry.

In *in vitro* experiments, we assessed the production of CA19-9 in pancreatic cancer cells and found that BxPC-3, AsPC-1, PK59, and HuCCT1 secreted substantial amounts of this molecule **(** **Figure 2A** **)**. Moreover, flow cytometric analysis also revealed that the amount of membrane-bound CA19-9 was high in cells that secreted CA19-9 (**Figure S1**). Membrane-bound CA19-9 could not be detected by ELISA in cell lines that do not secrete CA19-9 (**Figure S1**). Based on these results, we divided pancreatic cancer cell lines into two groups according to the level of CA19-9; i.e., CA19-9 high producer and non-producer cells.

We then investigated whether CA19-9-producing cancer cells could be targeted using these Fuc-Liposomes. Based on the results of preliminary experiments, we added Cy5.5 or FAM encapsulated L-fucose-liposomes (Fuc-Liposome-Cy5.5, Fuc-Liposome-FAM) to CA19-9 producing or non-producing cancer cells to confirm specificity of delivery. As shown in **Figure 2B**, fluorescence microscopy revealed that F50-Fuc-Liposomes but not F0-Fuc-Liposomes effectively introduced FAM into the cytosol of BxPC-3. Furthermore, flow cytometric analysis showed that F50-Fuc-Liposomes but not F0-Fuc-Liposomes effectively introduced Cy5.5 into the cytosol of BxPC-3 AsPC-1, and PK59 cells that secreted abundant CA19-9 within 2 hours (**Figure 2C and 2D** **, Figure S2**). Excess L-fucose inhibited the uptake of Cy5.5 into the CA19-9 producing cells but no remarkable change in CA19-9 non-producing cells, suggesting L-fucose specific introduction. Moreover, the amount of Cy5.5 transferred into pancreatic cancer cells seemed to increase in direct proportion to the level of CA19-9 expression (**Figure 2C** **, and Figure S2A**). Excess L-fucose but not D-glucose, D-mannose, D-xylose, or D-galactose decreased the efficiency of this process (**Figure 2E**), indicating that introduction of Cy5.5 by Fuc-Liposomes is indeed L-fucose dependent.

### Receptor Mediated-uptake of Fuc-Liposome into the Cells

**Figure 3:**
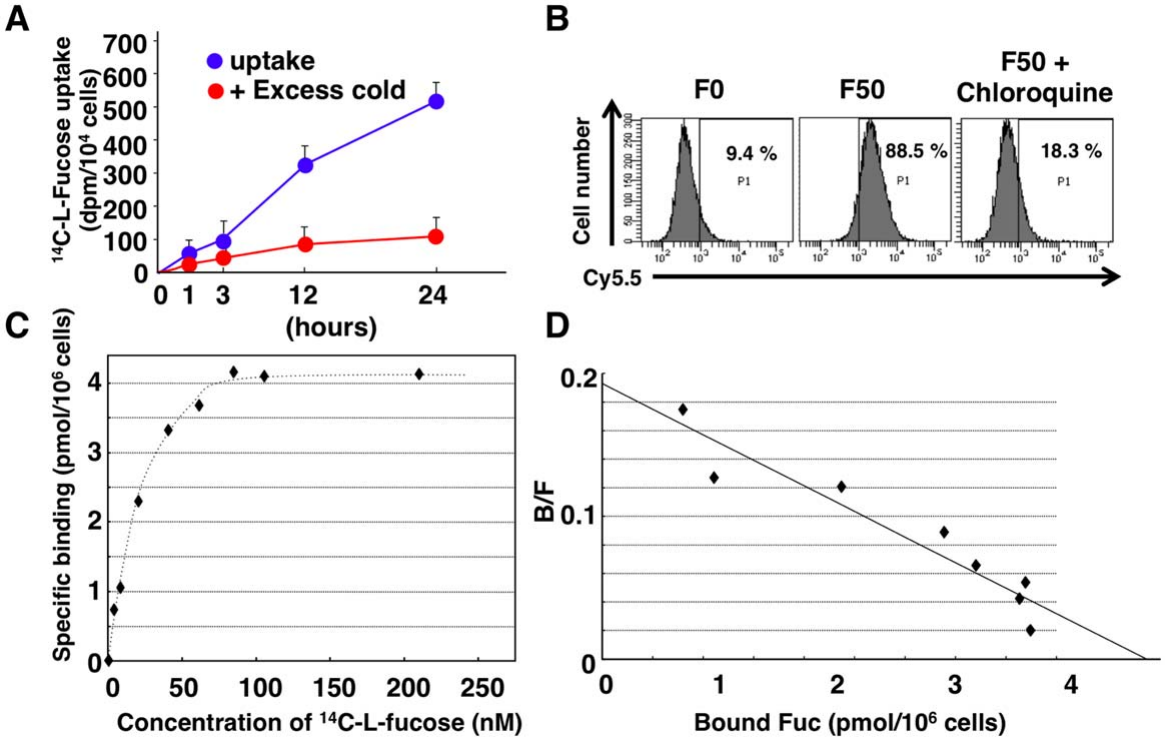
Receptor-mediated uptake of Fuc-Liposomes by pancreatic cancer cells. (**A**) Incorporation of ^14^C-labeled-L-fucose in AsPC-1 cells. Cells were incubated in the presence or absence of excess L-fucose (excess cold) in the ^14^C-labeled-L-fucose-containing medium for the indicated time, then ^14^C-labeled-L-fucose incorporation was measured. (**B**) BxPC-3 cells were incubated with or without chroloquine for 24 hours, treated with F50-Liposome-Cy5.5 for 2 hours at 37°C, and then analyzed by flow cytometry. (**C, D**) ^14^C-labeled-L-fucose binding assay using AsPC-1 cells. Scatchard plot analysis revealed 3.25×10^6^ receptors/cell, a K_d_ of 28.74 nM, and a Bmax of 5.49 pmol/10^6^ cells. Methods are described in *Materials and Methods*.

To verify L-fucose dependent uptake of Fuc-Liposome, the uptake of ^14^C-labeled L-fucose by AsPC-1 was examined. Incorporation of ^14^C-labeled-L-fucose in AsPC-1 was increased in a time-dependent manner, and was inhibited in the presence of excess cold L-fucose (**Figure3A**), suggesting the presence of L-fucose–specific binding protein. Inhibition of endocytosis by chroloquine led to suppression of Cy5.5 uptake in BxPC-3 cells (**Figure 3B**). We then performed a ^14^C-L-fucose receptor binding assay using AsPC-1 cells and detected a high affinity L-fucose–specific receptor (3.25×10^6^ receptors/cell, Kd  = 28.74 nM), indicating that uptake of L-fucose is mediated by its receptors (**Figure 3C, 3D**).

### Effect of Fuc-Liposome-Cisplatin on the Growth of Pancreatic Cancer Cell Lines

**Figure 4:**
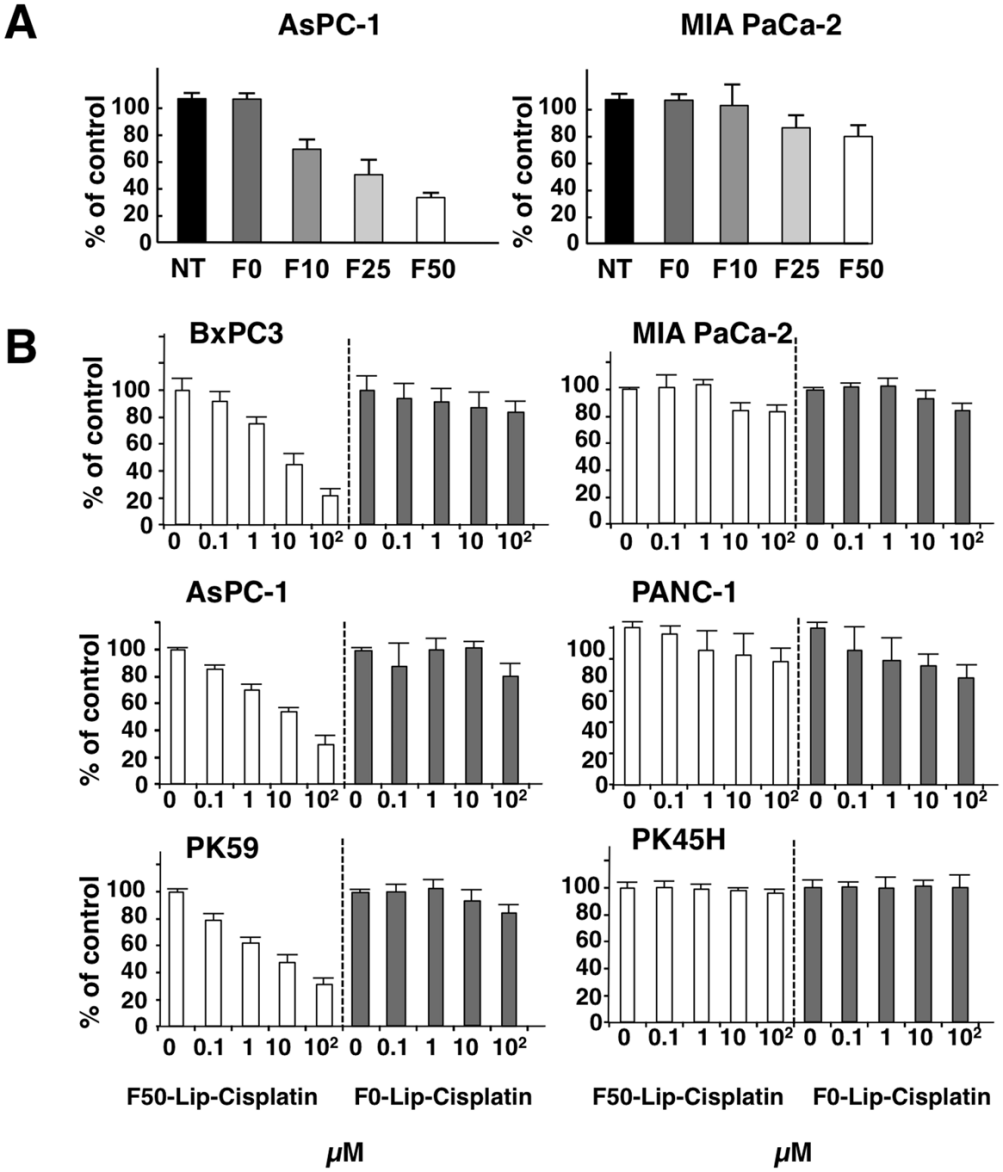
Effect of Fuc-Liposome-Cisplatin on the growth of pancreatic cancer cell lines. (**A, B**) Cells were treated with Fuc-Liposome containing Cisplatin for 2 hours, then washed and incubated for 72 hours. Viable cells were measured by WST assay.

We then encapsulated Cisplatin in Fuc-Liposomes. Fuc-Liposome-Cisplatin particles were approximately 200 nm in size, and the final concentration of Cisplatin was estimated at 2 mg/ml (**Figure S3 and Table S3**). This size of nanoparticle should allow penetration through the smallest capillary pores within the cancer vasculature by EPR effects, but should not breach the blood-brain barrier[16]. Cytotoxicity of Fuc-Liposome-Cisplatin was tested using the WST-1 assay (**Figure 4**). Pancreatic cancer cells were exposed to Fuc-Liposome-Cisplatin or Liposome-Cisplatin for 2 hours and then washed twice with phosphate-buffered saline to test the efficacy and specificity of Cisplatin transfer into CA19-9-producing cells. Because we observed the greatest cytotoxicity using F50-Liposome-Cisplatin (50 µg/ml Fuc-Liposomes) (**Figure 4A**), we selected this condition for the following experiments. In CA19-9-producing cells (BxPC-3, AsPC-1, PK59), F50-Liposome-Cisplatin exerted more potent effects than control liposomes (F0-Liposome-Cisplatin), indicating fucose-dependent cytotoxicity (**Figure 4B**). In addition to the effects on pancreatic adenocarcinoma cell lines, the growth of other cancers, such as the gastric and CRC cell line Colo205, which produce CA19-9, was also effectively suppressed by F50-Liposome-Cisplatin, indicating the potential applicability of this nanoparticle technology for the treatment of different types of cancer (**data not shown**). No cytotoxic effects of this agent were observed in non-CA19-9 producing cells (MIA PaCa-2, PANC-1, PK45H). Moreover, no cytotoxicity in normal cells such as peripheral blood mononuclear cells, fibroblasts, human umbilical vein endothelial cells (HUVEC)**,** or primary keratinocytes was seen, probably due to their low requirement for L-fucose (**Figure S4**). Because blood group antigens are determined by the pattern of glycoproteins, including molecules with attached L-fucose groups molecules expressed on the erythrocyte membrane, we were concerned that erythroblast precursors might also be inhibited. Therefore, we examined CFU-E/BFU-E colony formation by CD34+ cells in the presence or absence of F50-Liposome-Cisplatin. However, colony formation was not inhibited, regardless of the blood group of the CD34+ cells tested (**Figure S5**).

### Administration of D-mannose Augments Distribution of Fuc-Liposomes in CA19-9 Producing Tumors in a Xenograft Model

**Figure 5:**
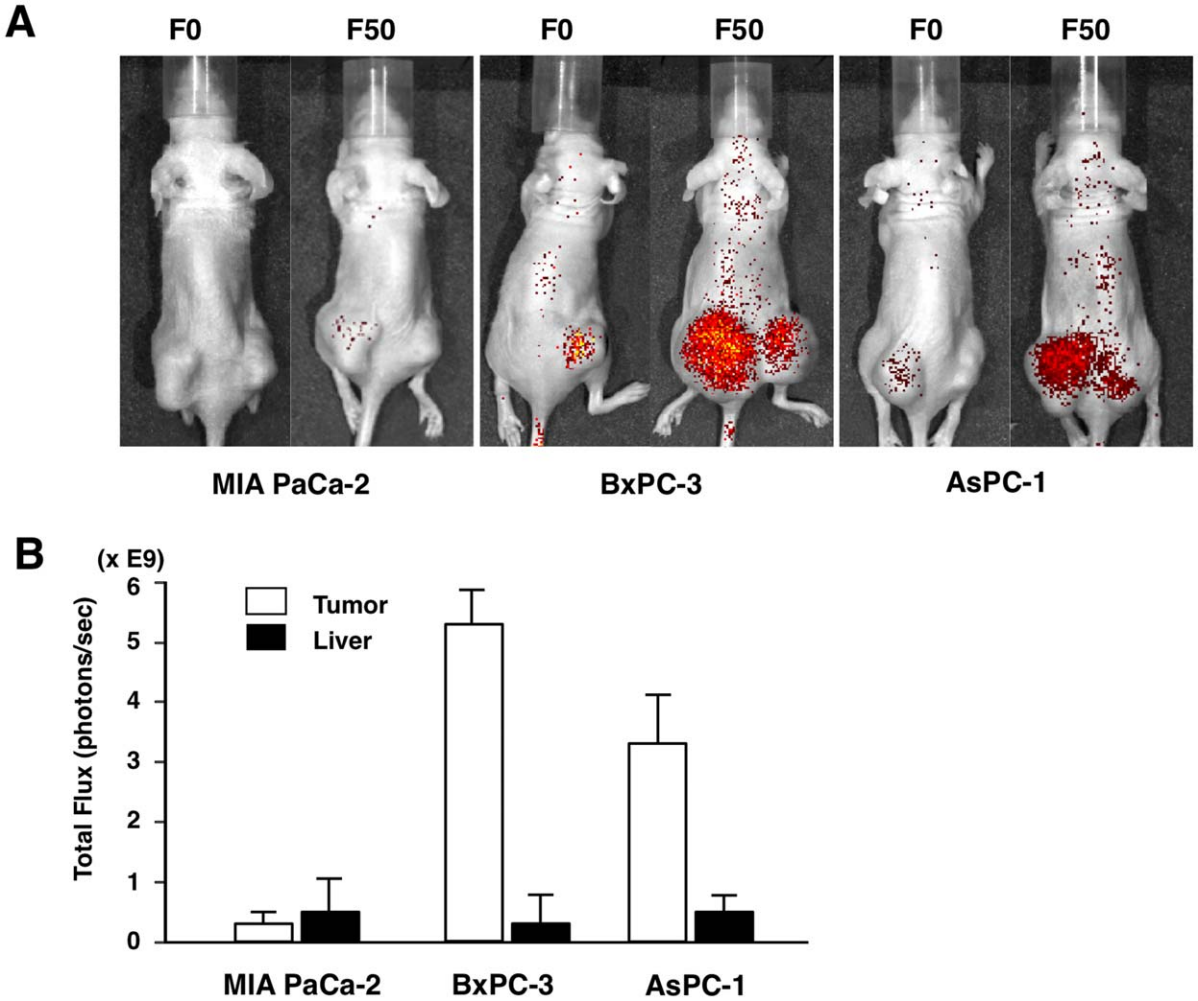
Pretreatment with D-mannose does not affect accumulation of F50-Liposome-Cy5.5. (**A**) Fuc-Liposome- or Liposome-Cy5.5 was administered via the tail vein (50 µl/mouse). The tumor regions of MIA PaCa-2, BxPC-3 and AsPC-1 cells (the back side of a bilateral flank lesion) in the mouse was observed and Cy5.5 accumulation was quantified using the IVIS imaging system at 96 hours after injection. D-mannose (1000-fold of the L-fucose) was injected simultaneously with liposome injection. (**B**) Total flux of the tumor and liver was calculated by using Living Image software according to the manufacturer’s instructions.

We next investigated the tumor-specific delivery of Fuc-Liposomes in tumor-bearing mice *in vivo*. It has been shown that clearance of L-fucose is delayed in D-mannose receptor-deficient mice [40], consistent with the presence of mannose/fucose receptors in the liver and Kupffer cells [41]–[44]. Furthermore, mannose-bound liposomes accumulated in non-parenchymal cells and Kupffer cells, when administered via the tail vein [45]. Based on these reports, we simultaneously administered D-mannose with Fuc-Liposomes in tumor-bearing mice to inhibit uptake of L-fucose via the D-mannose receptor. First, we tested the effect of D-mannose on the efficiency of Fuc-mediated uptake using flow cytometry. As shown in **Figure 2** **,** we confirmed that Fuc-liposomes were efficiently introduced into BxPC-3 cells *in vitro* even in the presence of excess D-mannose. Thereafter, we administered Fuc-liposomes *in vivo* and observed an accumulation of Cy5.5 in the tumor but reduction in the liver when D-mannose was administered before Fuc-Liposome injection (**Figure 5A and 5B** **, Figure S6**). Furthermore, accumulation of Cy5.5 was only observed in the CA19-9 producing tumor cells, BxPC-3 and AsPC-1, but not in CA19-9 non-producing tumor cells, (i.e., MIA PaCa-2). Accumulation of Cy5.5 in the tumor was sustained up to 1 week after Fuc-Liposome administration (**data not shown**) and no apparent side effects were observed.

### Fuc-Liposomes Carrying Cisplatin Suppressed Tumor growth and Prolonged Survival of Mice in the Xenograft Model

**Figure 6:**
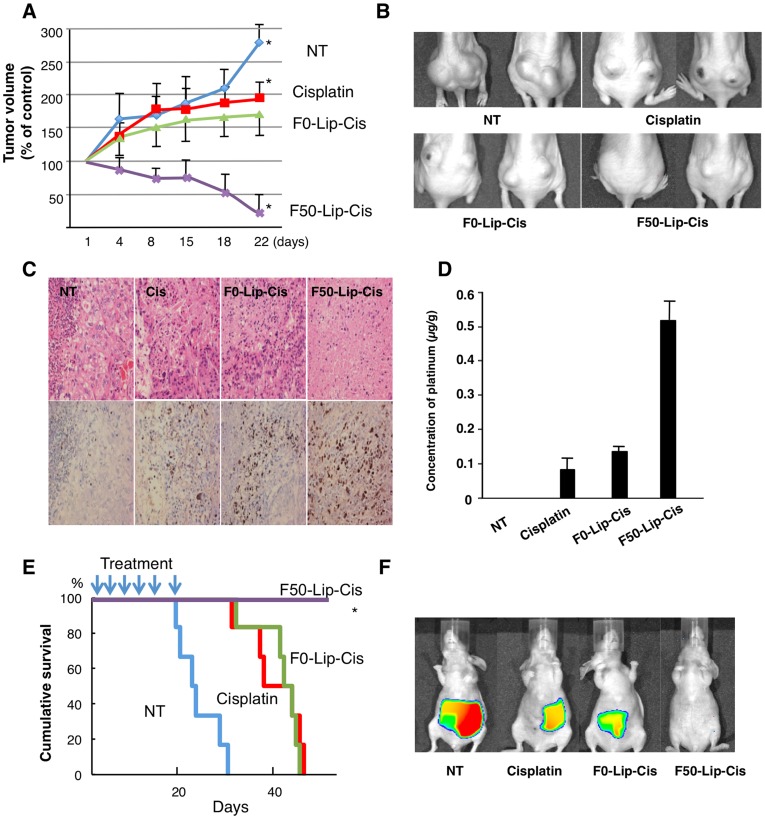
Fuc-liposomes carrying Cisplatin suppressed tumor growth and prolonged survival of mice in the xenograft model. (**A, B**) Comparison of tumor growth suppression with Cisplatin, F0-Liposome-Cisplatin, and F50-Liposome-Cisplatin in AsPC-1-bearing mice. Cisplatin (2 mg/kg), F0-Liposome-Cisplatin (2 mg/kg), or F50-Liposome-Cisplatin solution (2 mg/Cisplatin/kg) was injected via the tail vein of AsPC-1-bearing mice twice a week. At 4, 8, 11, 15, 18**,** and 22 days after transplantation, tumor volumes were measured. Representative image of mice treated with Cisplatin (**B**). Results are expressed as the mean ± SD (n = 6). *P<0.01 compared with NT, Cisplatin, and F0. (**C**) Tumor tissue was prepared on day 22 after treatment. HE staining (upper panel) and TUNEL staining (lower panel) are presented. (**D**) Concentration of platinum in the tumor tissue measured by ICP. (**E**) Survival rate of the mice treated with Cisplatin alone, F0-Liposome-Ciplatin, and F50-Liposome-Ciplatin in the liver metastasis model using BxPC-3 cells. Statistical analysis was performed by generalized Wilcoxon test. *P<0.01 compared with NT, Cisplatin, and F0. (**F**) Localization of BxPC3-Luc cells in the orthotopic model detected using an IVIS imaging system after 3 weeks of treatment.

In order to test the effects of Fuc-Liposome-Cisplatin on tumor growth *in vivo*, we developed a pancreatic adenocarcinoma xenograft model in mice. These animals were treated with Fuc-Liposome-Cisplatin twice a week. Tumor growth was significantly inhibited by treatment with F50-Liposome-Cisplatin compared with no treatment, F0-Liposome-Ciplatin, or Cisplatin alone, suggesting that F50-Fuc-Liposome could deliver Cisplatin specifically and efficiently (**Figure 6A and 6B**). In HE staining of tumor tissues, many viable cells were observed in untreated mice. The number of tumor cells decreased in Cisplatin-treated and F0-Liposome-Cisplatin treated-mice as compared with untreated mice. However, in mice treated with F50-Liposome-Cisplatin, tumor cells almost completely disappeared. TUNEL staining revealed the presence of greater numbers of apoptotic cells in tumors treated with F50-Liposome-Cisplatin than in controls (**Figure 6C**), possibly due to more marked accumulation of Cisplatin in the tumor tissue (**Figure 6D**). We also tested the effects of Fuc-Liposome-Cisplatin on survival in an orthotopic and liver metastasis model *in vivo* using BxPC-3-Luc cells. As shown in **Figure 6E** **,** survival of mice treated with Fuc-Liposome-Cisplatin was significantly prolonged relative to untreated mice, or relative to mice treated with Cisplatin alone or F0-Liposome-Ciplatin. In the orthotopic model, the tumor size in mice treated with Cisplatin alone or F0-Liposome-Cisplatin was nearly the same size but the tumors in both groups were smaller relative to untreated. Conversely, the tumor in mice treated with Fuc-Liposome-Cisplatin was not detected by *in vivo* imaging **(** **Figure 6F**).

No adverse effects, including body weight changes, attributable to the administration of either D-mannose or F50-Liposome-Cisplatin were observed during this study (**Table S4**).

## Discussion

We have generated and characterized L-fucose-bound liposomes containing Cisplatin, and have shown that Fuc-Liposome-Cisplatin is far more effective than Cisplatin alone in inhibiting proliferation of CA19-9-producing cancer cells *in vitro* and tumor growth *in vivo*. Pharmacokinetic experiments together with quantification of Cisplatin in targeted versus non-targeted delivery systems both in *vitro* and *in vivo* further confirmed that the inhibition of tumor growth was due to targeted delivery. Thus, our strategy utilizing biological characteristics of CA19-9 producing pancreatic cancer cells is promising with respect to specific cancer cell targeting.

We modified the liposome surface by coupling Tris via BS^3^, and cross-linking aminated L-fucose via DTSSP to achieve sufficient stealth and targeting function (**Figure 1A**). Particle size distribution remained stable after storage at 4°C for 6 months. Our liposomes are able to carry various kinds of drugs that are commonly used for cancer treatment, such as doxorubicin, oxaliplatin, and CPT-11 (**data not shown**).

While it has been reported that the postoperative survival rate is significantly worse in adenocarcinoma patients whose CA19-9 levels are markedly elevated [28], our results indicated that these patients with pancreatic cancers expressing CA19-9 would be appropriate candidates for treatment with Fuc-liposomes carrying anticancer drugs.

Fucose is utilized physiologically for fucosylation of glycoproteins in many cells types, especially in the liver via mannose/fucose receptors, which are abundantly expressed therein [41]–[43]. In preliminary experiments, we observed high accumulation of Cy5.5 probably through non-parenchymal cells and Kupffer cells. Nonetheless, we succeeded in maintaining liposome accumulation by administering mannose via the tail vein, which resulted in tumor targeting and preclinical confirmation of safety for potential clinical application [45].

In *Escherichia coli*, the cellular uptake of L-fucose is mediated by major facilitator family proton symporter, FucP [46]. Recently, the structure of the L-fucose transporter was identified in *E. coli* [47]. However, in humans, the mechanism of L-fucose cellular uptake is still controversial, with both diffusion and the active transport system proposed as major pathways for uptake of L-fucose into the cell [31]. Our Fuc-liposomes penetrate into the CA19-9-producing cells within 10 minutes and are inhibited by excess L-fucose, indicating the existence of a transporter or internalization system mediated by specific receptors on the cells, rather than merely a non-specific diffusion system. Indeed, receptor-binding assays revealed L-fucose–specific high affinity receptors on AsPC-1 cells (**Figure 3**). However, further investigation is required to resolve this issue.

Adverse effects on hematopoiesis, especially on red blood cell production, were a major concern with the use of Fuc-liposomes carrying anticancer drugs, but there was no change in either colony formation *in vitro* or bone marrow suppression *in vivo* during treatment. Although these adverse effects will also need to be screened for when using Fuc-liposomes carrying anti-cancer drugs other than Cisplatin, such as doxorubicin, we suspect that, given the limited requirement of normal cells for L-fucose or its limited delivery via normal blood vessels, the EPR effect and toxicity to bystander cells would be minimal.

The present report is the first to describe that targeted delivery of a cytotoxic drug as an L-fucose nanoconjugate can efficiently inhibit *in vivo* growth of pancreatic cancer cells. Furthermore, the L-fucose nanoparticles that we developed can be exploited as delivery vehicles for other anticancer drugs. This strategy could be extended as a generalized approach for the treatment of a wide variety of other CA19-9-producing carcinomas, such as colorectal (∼80% CA19-9 positive), biliary tract (70∼80% positive), and gastric carcinoma (20∼50% positive) [48], in addition to pancreatic adenocarcinoma (∼80% positive) [26], [27]. In conclusion, Fuc-liposome containing anticancer drugs should provide a new strategy for active targeting cancer therapy.

## Materials and Methods

### Materials

Cis-diammineplatinum (II) dichloride (Cisplatin), potassium tetrachloroplatinate(II), potassium iodide, ammonia aqueous solution (28%), silver nitrate, dipalmitoylphosphatidyl choline (DPPC), cholesterol (Chol), dicetylphosphate (DCP), sodium cholatehydrate (cholic acid), human serum albumin (HSA), sodium periodate, deuterium oxide (D_2_O), sodium hexachloroplatinate, tris(hydroxymethyl)aminomethane (Tris), and L-fucose were purchased from Sigma (St. Louis, MO, USA). Ganglioside was purchased from Avanti Polar Lipids (Alabaster, AL, USA). Dipalmitoylphosphatidylethanolamine (DPPE) was purchased from Alexis (Plymouth Meeting, PA, USA). N-tris(hydroxymethyl)methyl-3-amino-propane sulfonic acid (TAPS) and n-(2-hydroxyethyl) piperazine-n’-(2-ethanesulfonic) acid were purchased from Dojin Chemical (Kumamoto, Japan). Sodium cyanoborohydrate was purchased from Aldrich (Milwaukee, WI, USA). Bis(sulfosuccinimidyl)suberate (BS^3^) and 3,3-dithiobis(sulfosuccinimidylpropionate) (DTSSP) were purchased from Pierce Biotechnology (Rockford, IL, USA). Cholesterol E-test Wako was purchased from Wako (Osaka, Japan). Potassium dichloroplatinum was purchased from Nacalai Tesque (Kyoto, Japan). Chroloquine was purchased from Sigma.

### Preparation of Cy5.5, FAM and Cisplatin Encapsulated in Liposomes

See Text S1 section.

### Cell Lines

The pancreatic cancer cell lines KP4, PK-59, PK-45H, MIA PaCa-2, PANC-1, and HuCCT1 were obtained from the Riken BRC Cell Bank. AsPC-1 and BxPC-3 were purchased from American Type Culture Collection. BxPC-3, AsPC-1, PANC-1, PK-45H, PK-59, and HuCCT1 cells were cultured in RPMI 1640 (Gibco) supplemented with 10% FBS, L-glutamine, and 1% penicillin-streptomycin. KP4 and MIA PaCa-2 were cultured in DMEM (Gibco) supplemented with 10% FBS, L-glutamine, and 1% penicillin-streptomycin. CD34+ cells were purchased from Takara Bio Inc. Normal human umbilical vein endothelial cells (HUVEC) were purchased from BD Bioscience and cultured in the medium supplied by the manufacturer. Normal human keratinocytes were purchased from Takara and cultured using the KGM-Gold™ Bullet Kit. Normal human fibroblasts were obtained from ATCC and cultured using the Fibroblast Growth Kit supplied by the cell provider.

### Flow Cytometric Analysis

Cells (1×10^5^ cells) treated with Fuc-Liposome-Cy5.5 or Liposome-Cy5.5 (lipid concentration: 4 µg/ml) were cultivated for 2 hours. For the blocking assay, 1×10^5^ cells were treated with L-fucose, D-mannose, D-glucose, D-xylose, D-galactose for 30 minutes prior to adding Fuc-Liposome-Cy5.5 or Liposome-Cy5.5. The MFI was assessed on a FACScalibur with CellQuest software (Becton Dickinson).

### Intracellular Distribution Analysis of Cy5.5 and FAM

Cells were plated in Lab-Tek chambered coverglasses at 1×10^4^ cells/chamber. Fuc-Liposome-Cy5.5 or Liposome-Cy5.5 was added to cells at a final Cy5.5 concentration of 0.7 µg/ml. In the case of Fuc-Liposome-FAM, the final concentration of FAM was adjusted to 4 ng/ml. Cells were cultured in complete medium for 30 minutes, after which the medium was replaced with fresh medium. At 30 minutes and 2 hours post-treatment, the cells were washed twice with PBS and fixed with 4% paraformaldehyde for 15 minutes at room temperature. After fixation, the cells were washed 3 times with PBS and exposed to Prolong Gold Antifade Reagent with DAPI (Molecular Probes) for 10 minutes to stain nuclei. The subcellular localization of Cy5.5 was assessed using confocal laser microscopy (Zeiss, Germany) and fluorescence microscopy (Keyence, BZ-8000).

### L-fucose Receptor Binding and Uptake Assay

For binding studies, cells were seeded at 1×10^5^ per well in 12-well culture plates. Cells were incubated with increasing concentrations (0.05 - 200 nM) of [^14^C]-labeled-L-fucose with or without a 200-fold excess of unlabeled L-fucose in 1.0 ml serum-free medium at 4°C for 1 hour with gentle agitation. Following incubation, cells were washed three times with PBS to reduce non-specific binding. Cells were harvested and the cell lysate was transferred to a scintillation vial. The apparent dissociation constant (Kd) was derived from Scatchard analysis, using linear regression to provide a best fit of the binding data.

### *In vitro* cell Proliferation Assay

See Text S1 section.

### Pancreatic Cancer Xenograft Model, Noninvasive Imaging, and Treatment Schedule

1. **Subcutaneous model.** In the subcutaneous model, 2×10^6^ cells were inoculated to create a dorsal lesion (mice aged 4 to 6 weeks) and were allowed to grow into tumors 5 mm in diameter. AsPC-1-bearing mice were treated with Cisplatin (2 mg/kg), F0-Liposome-Cisplatin (2 mg/kg), or F50-Liposome-Cisplatin solution (2 mg/Cisplatin/kg) via the tail vein twice a week. At 4, 8, 11, 15, 18**,** and 22 days after transplantation, tumor volumes were measured. Representative image of mice treated with Cisplatin. For D-mannose pre-treatment in all the *in vivo* experiment, 5 mg of D-mannose was injected through tail vein 5 minutes before administration of agents.
2. **Liver metastasis model.** In the case of the liver metastasis model, BxPC-3-Luc cells (3×10^6^) in 100 µl PBS were inoculated into the spleens of 4-week-old female nude mice (n = 8) through small incisions in the left lateral flank using a 1-ml syringe and 27-gauge needle. On day 5 after tumor inoculation, splenectomy was performed as described previously [49]. *In vivo* optical imaging for luciferase was done ∼20 minutes after i.p. injection of 3 mg n-luciferin into each animal using a Xenogen-IVIS–cooled CCD optical system (Xenogen-IVIS). The mice were randomized into no treatment, Cisplatin, F0-Lipsome-Cisplatin and F50-Liposome-Cisplatin groups. The administered dose (via the tail vein) of Cisplatin, either bound (as in Fuc-Liposome-Cisplatin) or free, was 2 mg/kg/dose. The injection schedule was twice in the first week, followed by once at week 2 and once at week 3. All mice were sacrificed on the day after the last injection but before a final bioluminescence measurement. In each group, two of the eight mice were sacrificed at day 43 after tumor inoculation for the measurement of liver weight and the number of metastatic foci on the liver surface. The remaining six mice in each group were kept for survival analysis.
3. **Orthotopic model.** In the orthotopic model, BxPC-3-Luc cells (3×10^6^) in 100 µl PBS were orthotopically injected into the pancreas of nude mice (ages 4 to 6 weeks) as described previously. On day 0–4 postinjection, bioluminescence was measured and the mice were randomized into different groups before the initiation of treatment. The injection schedule was twice in the first week, followed by once at week 2 and once at week 3. All mice were sacrificed on the day after the last injection but before a final bioluminescence measurement.

These studies were carried out with the recommendations in the Guide for the Care and Use of Laboratory Animals of the National Institutes of the Health. The protocols were approved by the Committee on the Ethics of Animal Experiments of Sapporo Medical University. All surgery was performed under sodium pentobarbital anesthesia, and all efforts were made to minimize suffering.

### Cisplatin Concentration in the Tumor Tissue

The day after the last injection, all mice were sacrificed and tumors were collected for platinum concentration assay, measured using ICP analysis.

### Statistics

Results are presented as means (± SD) for each sample. Differences between the two groups were examined by the unpaired *t* test and paired *t* test. If two groups could not be considered to be of equal variance, the *t* test with Welch’s correction was performed. Multiple comparisons between control groups and other groups were performed by Dunnet’s test.

Methods and any associated references are available in the online version of the paper.

## Supporting Information

Figure S1 **Flow cytometric analysis for CA19-9.** Pancreatic cancer cell lines (5×10^5^ cells) were incubated with isotypic control or CA19-9 antibody (AbCam) on ice for 30 minutes. After washing in PBS/0.05% BSA, the cells were incubated with FITC labeled anti-mouse goat IgG (R & D) for 30 minutes on ice. Cells were washed twice in PBS/0.05% BSA and analyzed by flow cytometry (Beckton Dickinson).
(TIFF)

Figure S2 **Transfer of Fuc-Liposome-Cy5.5 into pancreatic cancer cells. (A)** Flow cytometric analysis of Fuc-Liposome-Cy5.5-treated cells. BxPC-3, PK59, AsPC-1 (CA19-9 producing cancer cells) and PANC-1, PK45H, MIA PaCa-2, KP4 (CA19-9 non-producing pancreatic cancer cells) cells were treated with Fuc-Liposome-Cy5.5 for 2 hours with or without excess L-fucose and were analyzed by flow cytometry. NT, no treatment: F0, F0-Liposome-Cy5.5: F25, F25-Liposome-Cy5.5: F50, F50-Liposome-Cy5.5: F50+ Fuc, excess L-Fucose. **(B)** HuCCT1 (CA19-9 producing) cells were incubated with Fuc-Liposome-Cy5.5 for indicated hours in the presence or absence of excess L-fucose, then washed twice with phosphate-buffered saline and analyzed by flow cytometry.
(TIFF)

Figure S3 **Electron microscopic image of Fuc-Liposome-Cisplatin (F50).** Scale bar shows 50 nm.
(TIFF)

Figure S4 **Effect of Fuc-Liposome-Cisplatin on the growth of normal cells.** **(A, B)** Cells were treated with Fuc-Liposomes containing Cisplatin for 2 hours and then washed and incubated for 72 hours. Viable cells were quantified using the WST assay.
(TIFF)

Figure S5 **Effect of Fuc-liposome-Cisplatin on** **CFU-E/BFU-E colony formation.** CD34+ cells of known blood type were seeded in MethoCult-H4230 (StemCell Technologies) in the presence or absence of 1 µM Fuc-liposome-Cisplatin or Cisplatin alone, and cultured for 2 weeks. CFU-E/BFU-E colonies were counted, and colony numbers with no treatment were set as one hundred percent.
(TIFF)

Figure S6 **Inhibition of F50-Liposome-Cy5.5 accumulation in liver by pretreatment with D-mannose.** *In vivo* image of tumor (AsPC-1) bearing mice treated with F50-Liposome-Cy5.5. Tumor bearing mice were treated with F50-Liposome-Cy5.5 after administration of D-mannose, then after 48 hours, Cy5.5 was visualised by *In vivo* image analyser.
(TIFF)

Table S1 **Physicochemical characteristics of Cy5.5 liposomes.**
(TIFF)

Table S2 **Physicochemical characteristics of FAM liposome.**
(TIFF)

Table S3 **Physicochemical characteristics of Cisplatin liposomes.**
(TIFF)

Table S4 **Hematological and non-hematological adverse effects during Fuc-Liposome-Cisplatin administration.**
(TIFF)

Text S1 **This file includes Supporting Materials and Mehods with references.**
(DOC)
