# Supplementary figures and images for: Targeting Anticancer Drug Delivery to Pancreatic Cancer Cells Using a Fucose-Bound Nanoparticle Approach
Source: PLoS One. 2012 Jul 11;7(7):e39545. doi: 10.1371/journal.pone.0039545 (PMC3394772; doi:10.1371/journal.pone.0039545)

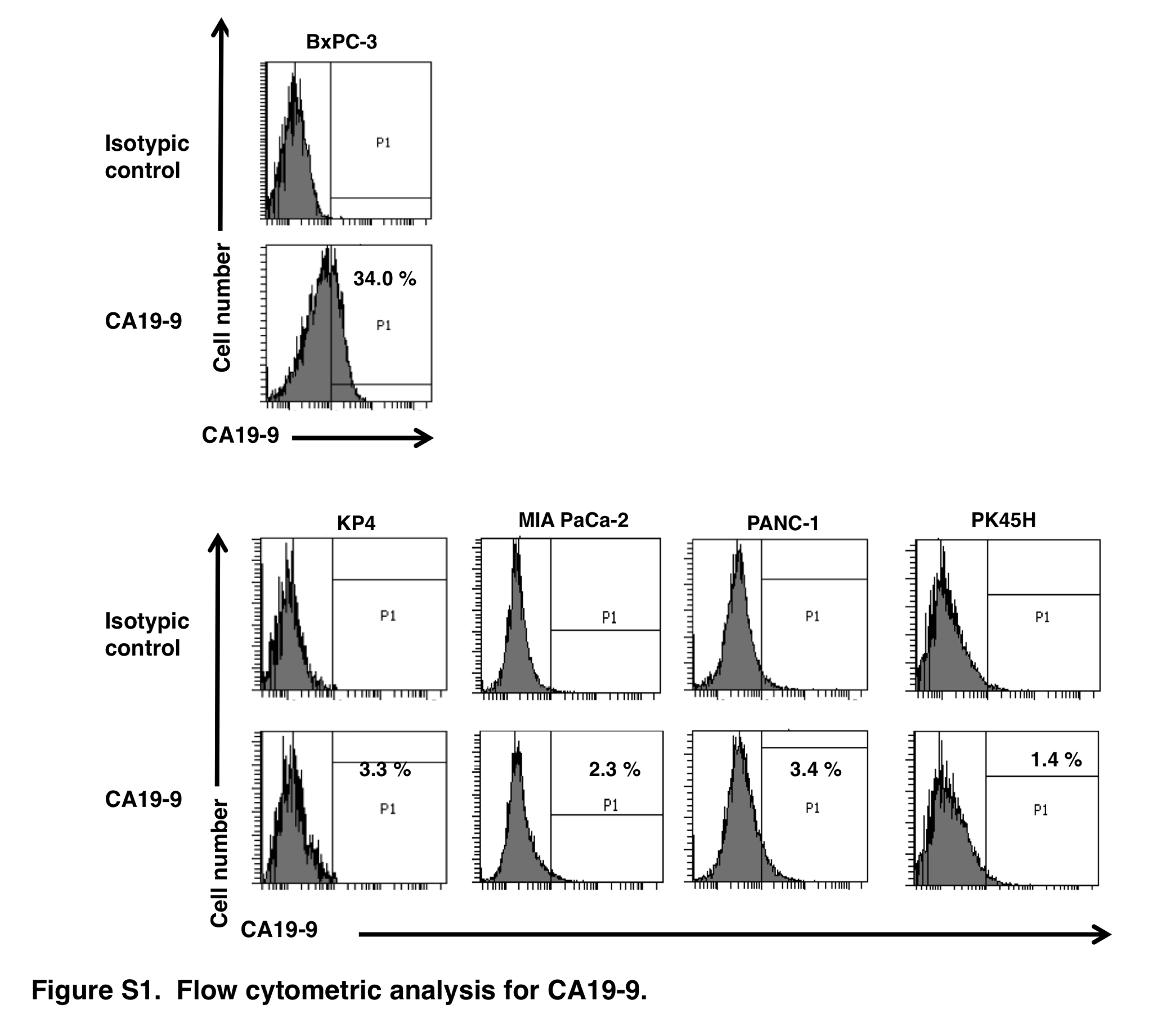

Supplement: Figure S1 — Flow cytometric analysis for CA19-9. Pancreatic cancer cell lines (5×10^5^ cells) were incubated with isotypic control or CA19-9 antibody (AbCam) on ice for 30 minutes. After washing in PBS/0.05% BSA, the cells were incubated with FITC labeled anti-mouse goat IgG (R & D) for 30 minutes on ice. Cells were washed twice in PBS/0.05% BSA and analyzed by flow cytometry (Beckton Dickinson). (TIFF) [file pone.0039545.s001.tif]

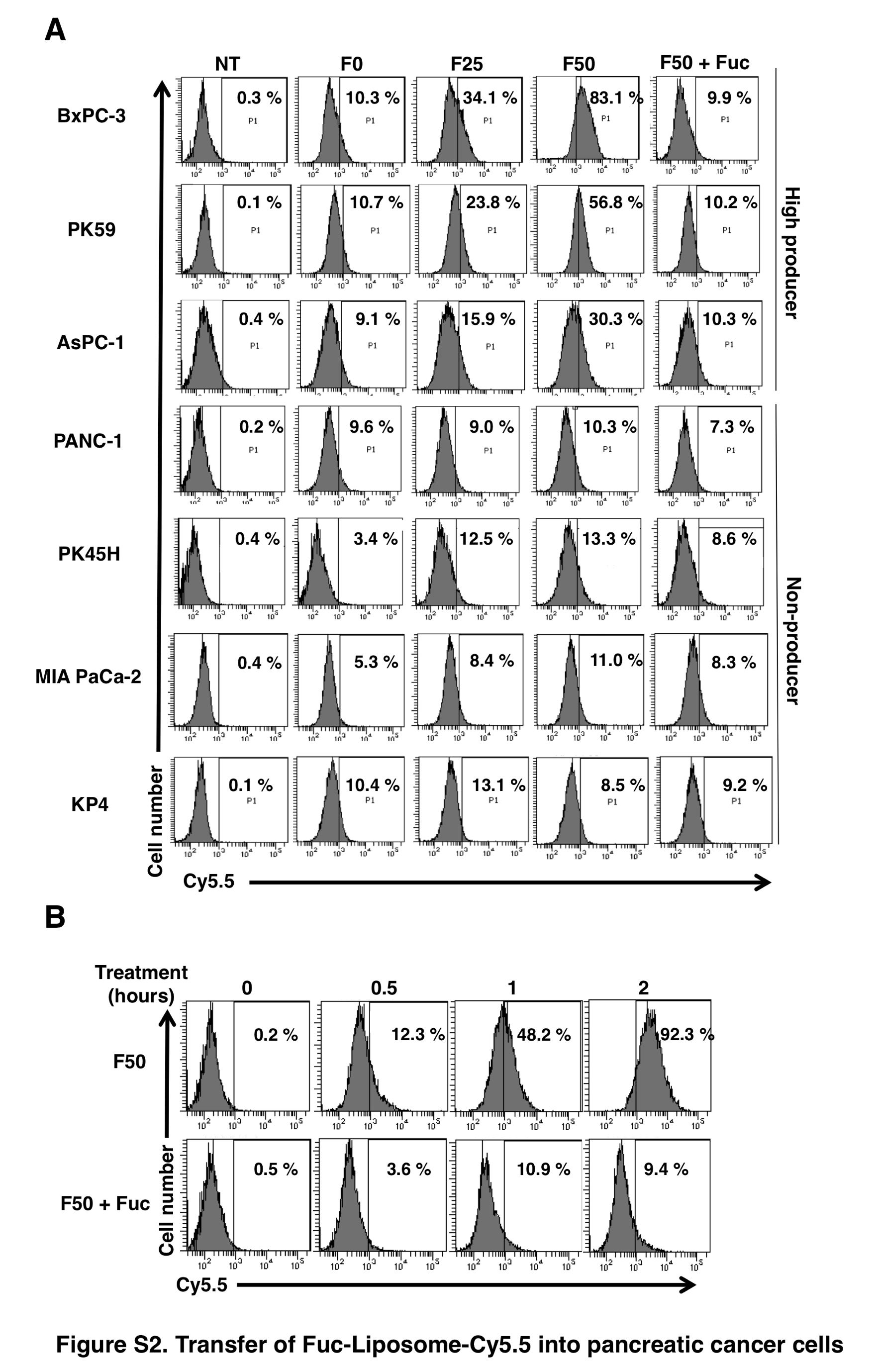

Supplement: Figure S2 — Transfer of Fuc-Liposome-Cy5.5 into pancreatic cancer cells. (A) Flow cytometric analysis of Fuc-Liposome-Cy5.5-treated cells. BxPC-3, PK59, AsPC-1 (CA19-9 producing cancer cells) and PANC-1, PK45H, MIA PaCa-2, KP4 (CA19-9 non-producing pancreatic cancer cells) cells were treated with Fuc-Liposome-Cy5.5 for 2 hours with or without excess L-fucose and were analyzed by flow cytometry. NT, no treatment: F0, F0-Liposome-Cy5.5: F25, F25-Liposome-Cy5.5: F50, F50-Liposome-Cy5.5: F50+ Fuc, excess L-Fucose. (B) HuCCT1 (CA19-9 producing) cells were incubated with Fuc-Liposome-Cy5.5 for indicated hours in the presence or absence of excess L-fucose, then washed twice with phosphate-buffered saline and analyzed by flow cytometry. (TIFF) [file pone.0039545.s002.tif]

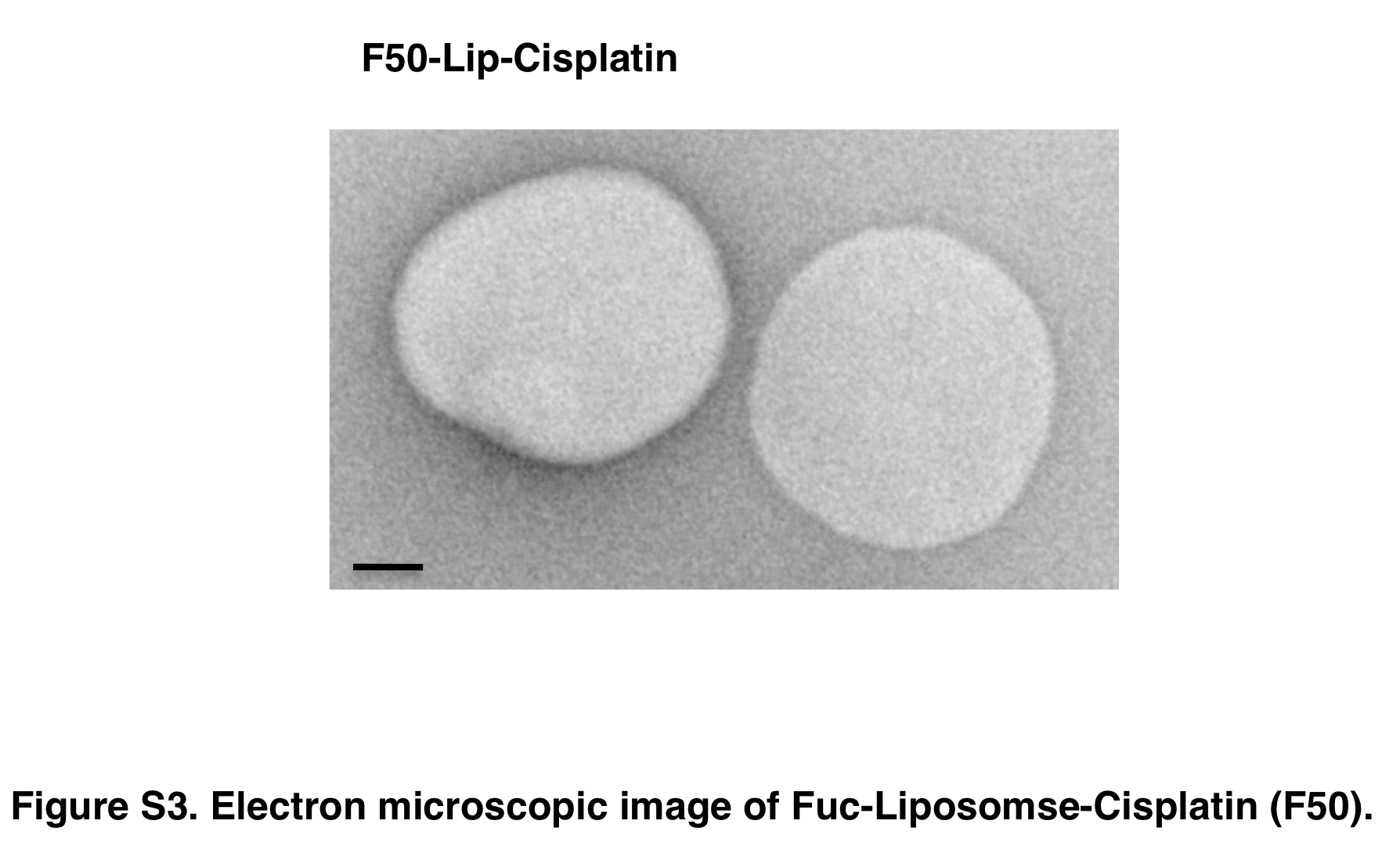

Supplement: Figure S3 — Electron microscopic image of Fuc-Liposome-Cisplatin (F50). Scale bar shows 50 nm. (TIFF) [file pone.0039545.s003.tif]

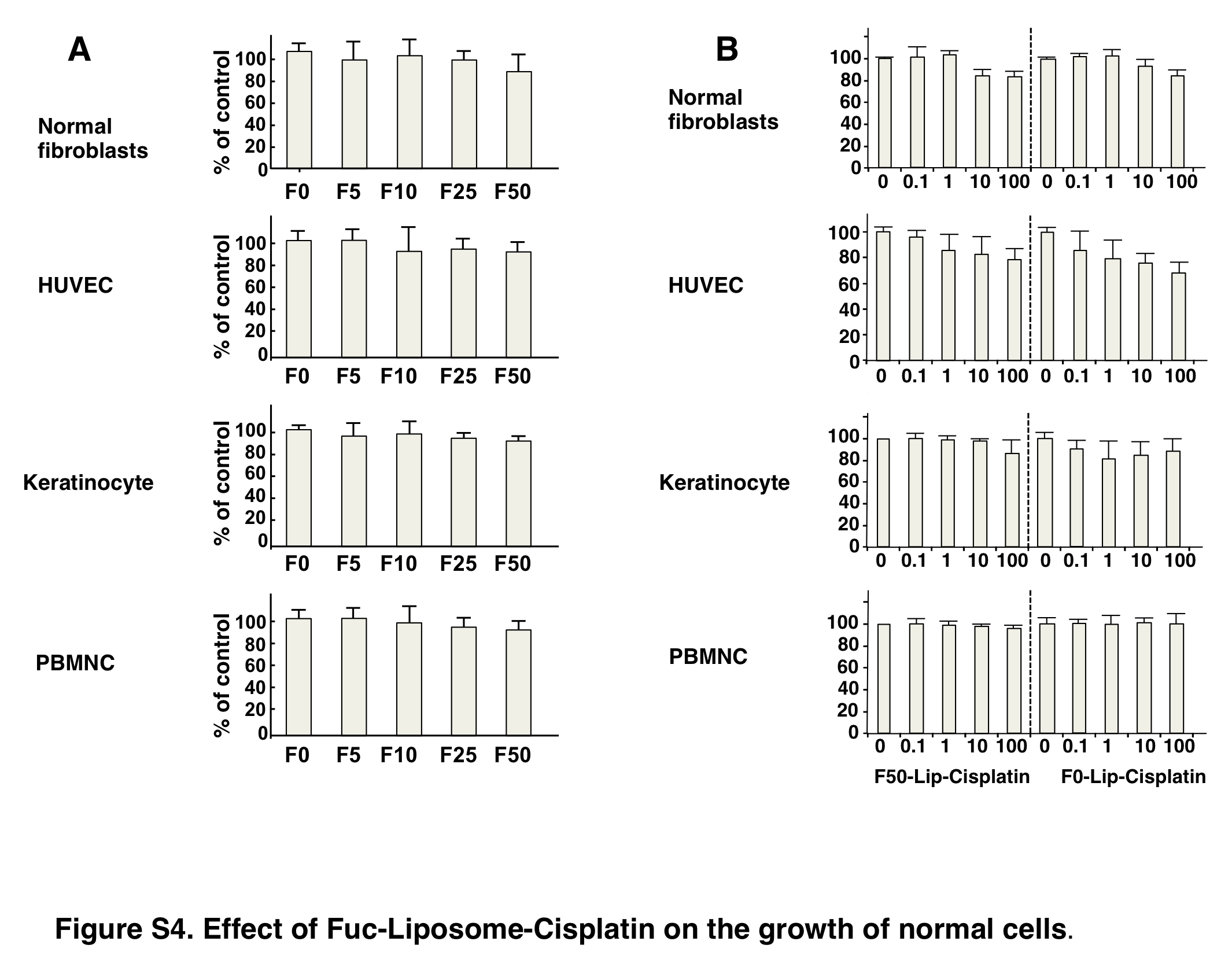

Supplement: Figure S4 — Effect of Fuc-Liposome-Cisplatin on the growth of normal cells. (A, B) Cells were treated with Fuc-Liposomes containing Cisplatin for 2 hours and then washed and incubated for 72 hours. Viable cells were quantified using the WST assay. (TIFF) [file pone.0039545.s004.tif]

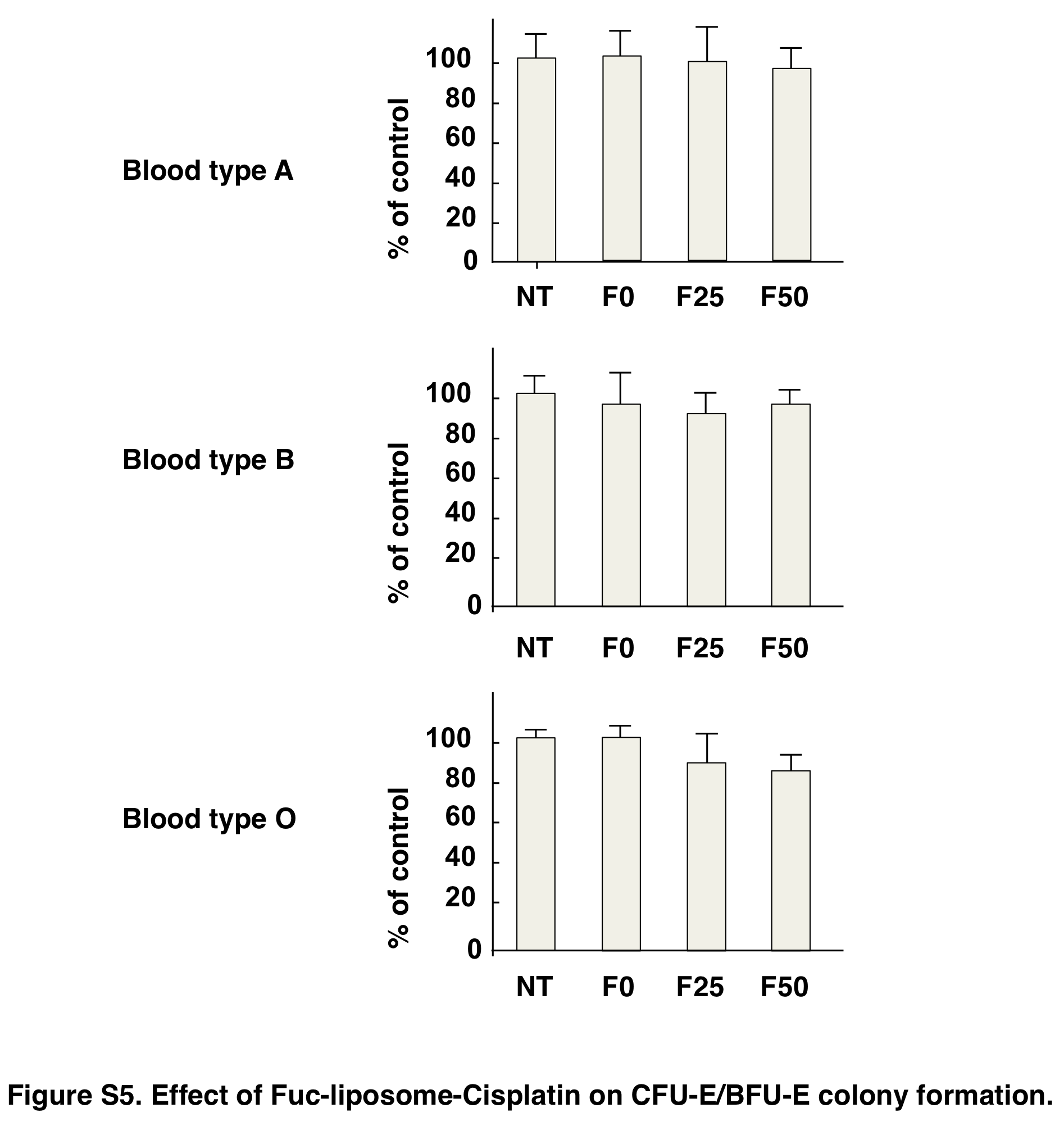

Supplement: Figure S5 — Effect of Fuc-liposome-Cisplatin on CFU-E/BFU-E colony formation. CD34+ cells of known blood type were seeded in MethoCult-H4230 (StemCell Technologies) in the presence or absence of 1 µM Fuc-liposome-Cisplatin or Cisplatin alone, and cultured for 2 weeks. CFU-E/BFU-E colonies were counted, and colony numbers with no treatment were set as one hundred percent. (TIFF) [file pone.0039545.s005.tif]

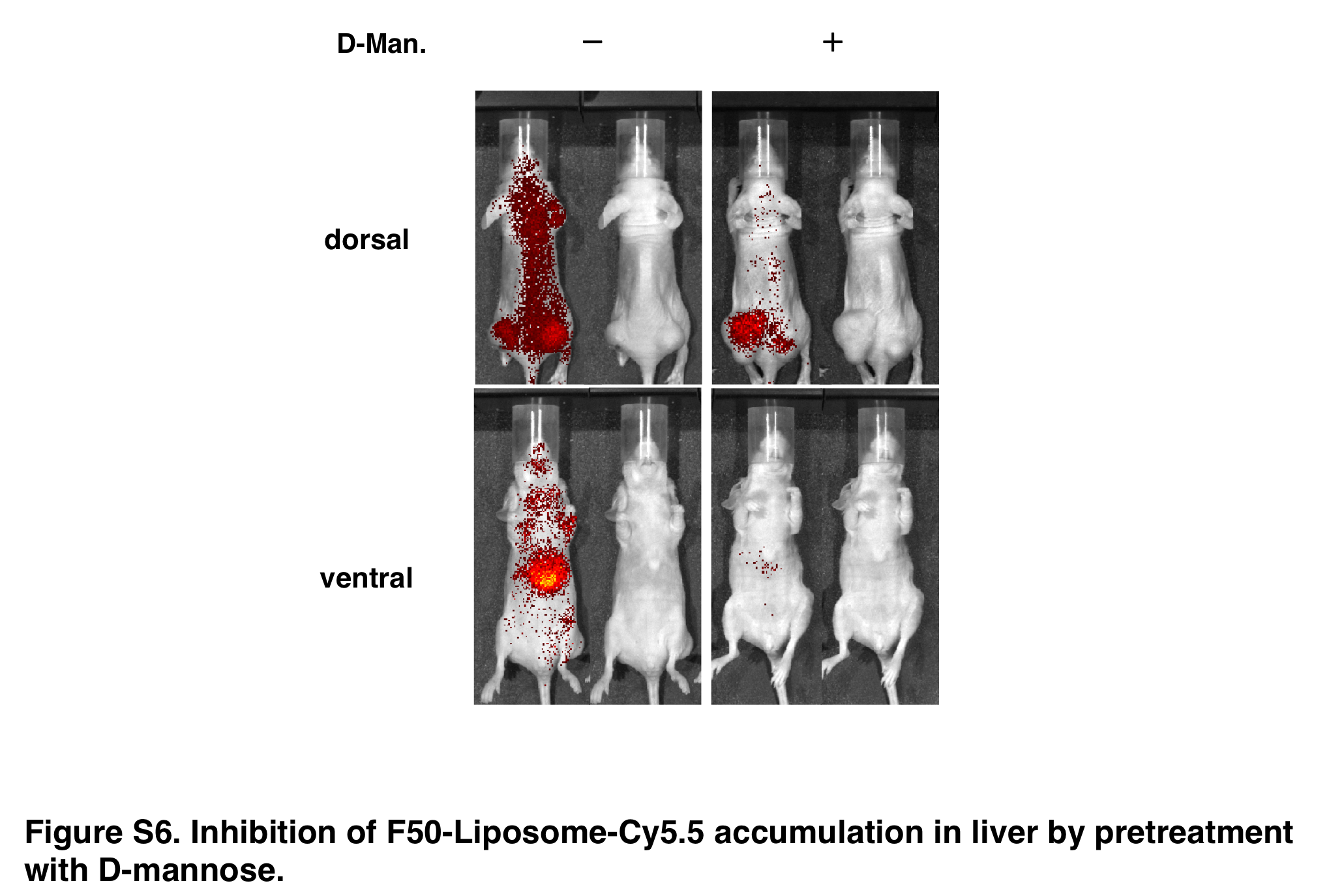

Supplement: Figure S6 — Inhibition of F50-Liposome-Cy5.5 accumulation in liver by pretreatment with D-mannose. In vivo image of tumor (AsPC-1) bearing mice treated with F50-Liposome-Cy5.5. Tumor bearing mice were treated with F50-Liposome-Cy5.5 after administration of D-mannose, then after 48 hours, Cy5.5 was visualised by In vivo image analyser. (TIFF) [file pone.0039545.s006.tif]

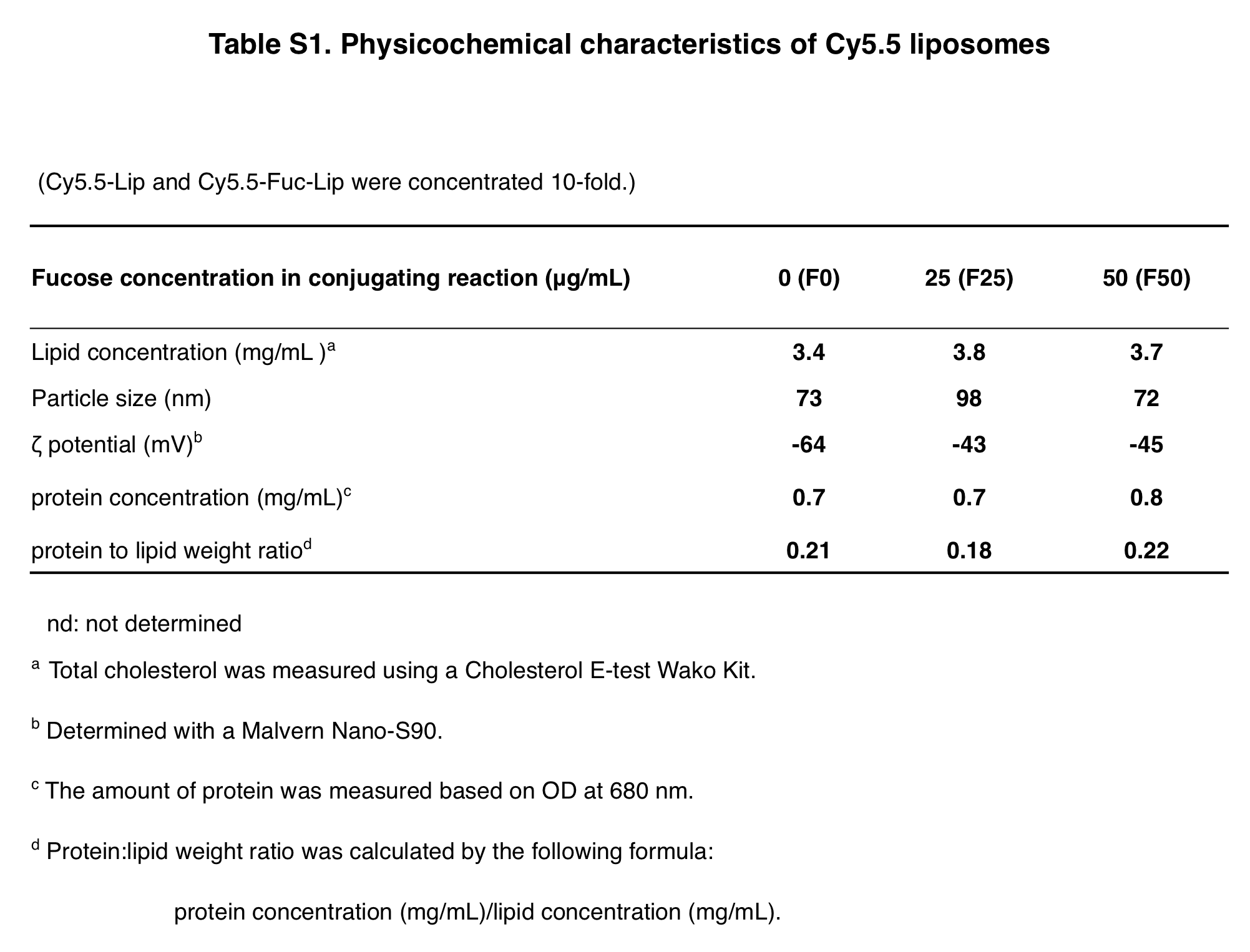

Supplement: Table S1 — Physicochemical characteristics of Cy5.5 liposomes. (TIFF) [file pone.0039545.s007.tif]

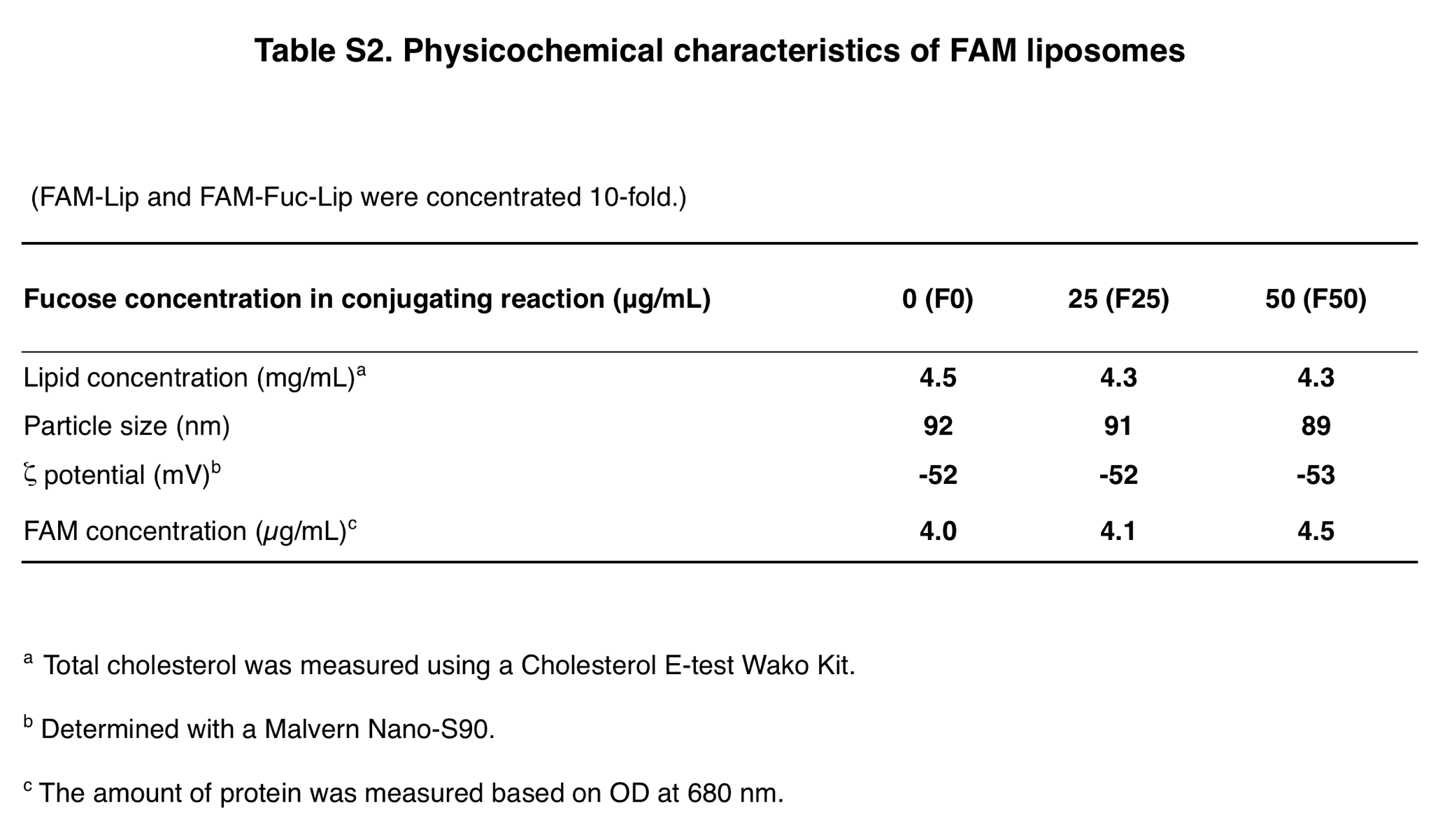

Supplement: Table S2 — Physicochemical characteristics of FAM liposome. (TIFF) [file pone.0039545.s008.tif]

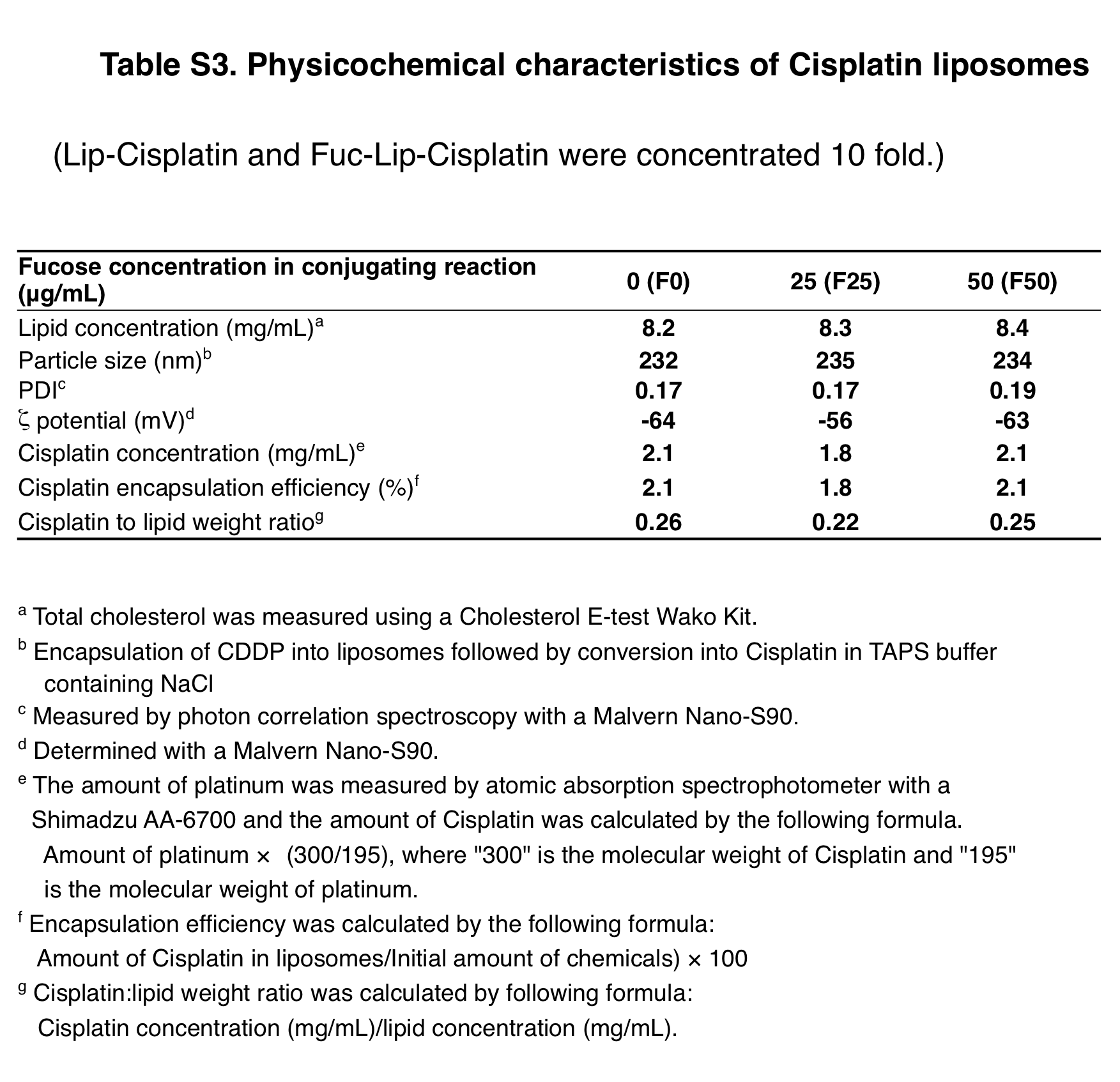

Supplement: Table S3 — Physicochemical characteristics of Cisplatin liposomes. (TIFF) [file pone.0039545.s009.tif]

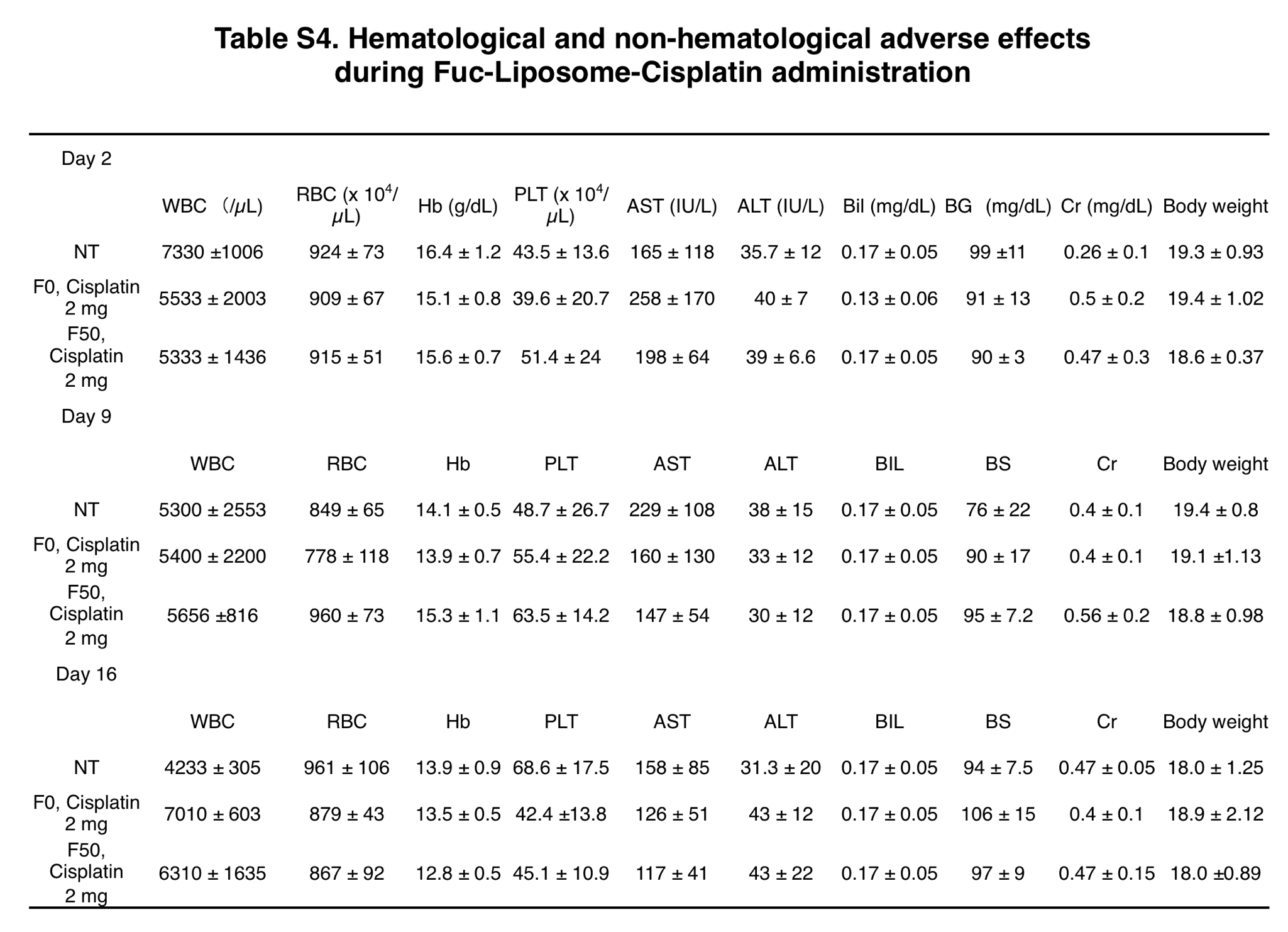

Supplement: Table S4 — Hematological and non-hematological adverse effects during Fuc-Liposome-Cisplatin administration. (TIFF) [file pone.0039545.s010.tif]
