# Supplementary material for: Targeting Anticancer Drug Delivery to Pancreatic Cancer Cells Using a Fucose-Bound Nanoparticle Approach
Source: PLoS One. 2012 Jul 11;7(7):e39545. doi: 10.1371/journal.pone.0039545 (PMC3394772; doi:10.1371/journal.pone.0039545)
Supplement: Text S1 — This file includes Supporting Materials and Mehods with references. (DOC) [file pone.0039545.s011.doc]

**Text S1**

**Supporting Materials and Methods**

**Targeting Anticancer Drug Delivery to Pancreatic Cancer Cells Using A Fucose-Bound Nanoparticle Approach**

Makoto Yoshida^1, 2^, Rishu Takimoto^1, 2^, Kazuyuki Murase^1^, Yasushi Sato^1^, Masahiro Hirakawa^1, 2^, Fumito Tamura^1, 2^, Tsutomu Sato^1, 3^, Satoshi Iyama^1^, Takahiro Osuga^1^, Koji Miyanishi^1^, Kohichi Takada^1^, Tsuyoshi Hayashi^1^, Masayoshi Kobune^3^, and Junji Kato^1, 2^

^1^Fourth Department of Internal Medicine, Sapporo Medical University School of Medicine, South-1, West-16, chuo-ku, Sapporo, 060-8543, Japan, ^2^Division of Clinical Oncology, ^3^Division of Molecular Oncology, Sapporo Medical University Graduate School of Medicine, South-1, West-16, chuo-ku, Sapporo, 060-8543, Japan

**Correspondence to**: Junji Kato, MD, PhD, Fourth Department of Internal Medicine, School of Medicine Sapporo Medical University, South-1, West-16, chuo-ku, Sapporo, 060-8543, Japan (e-mail: [jkato@sapmed.ac.jp](mailto:jkato@sapmed.ac.jp)).

Keywords: pancreatic cancer, cancer targeting, L-fucose, nanoparticle

Preparation of Cy5.5, FAM and Cisplatin encapsulated in liposomes

Briefly, DPPC, Chol, ganglioside, DCP and DPPE were mixed at different molar ratios and cholic acid was added to facilitate micelle formation. The mixture was dissolved in methanol/chloroform (1:1, v/v) and the solvent evaporated at 37°C to produce a lipid film, which was dried under vacuum. This was then dissolved in 10 mM TAPS buffer without NaCl at pH 8.4, and sonicated to obtain a suspension of uniform micelles. CDDP3^1^, Cy5.5, or FAM solution was then added to the micelle suspension, which was then ultrafiltered (molecular cut off 10,000) (Amicon PM10, Millipore, Billerica, MA, USA). The CDDP3 in the liposomes was then converted into Cisplatin. Hydrophilization treatment and L-fucose conjugation on the surface of liposomes were carried out by methods modified from Yamazaki et al^2^. Briefly, the crosslinking agent BS^3^ was added to the liposome solution, which was then stirred at 25°C for 2 hours and then overnight at 4°C. Then another 400 mg of BS^3^ was added and the mixture was incubated for 2 hours at 25°C and overnight again at 4°C to bind Tris to BS^3^. Human serum albumin was coupled to the liposome surface as previously described^2^. Sodium periodate was used to oxidize the liposome surface. To remove residual sodium periodate, the suspension was ultrafiltered with 10 mM phosphate buffered saline (PBS, pH 8.0) through an Amicon XM300 membrane. HSA was then added to the suspension and stirred at 25°C for 2 hours before addition of sodium cyanoborohydrate and incubation for 2 addiitonal hours at 25°C and overnight at 4°C. L-fucose (2 mg) was dissolved in 0.5 ml of distilled water. Then 0.25 g of NH_4_HCO_3_ was added and stirred at 37 °C for 3 days to yield aminated L-fucose according to the method of Zeng et al^3^. Aminated L-fucose was conjugated to the liposome surface through DTSSP in a similar manner. DTSSP (100 mg), a crosslinking agent, was added to 100 ml of liposome solution, and stirred at 25°C for 2 hours, and further overnight at 4°C. To remove residual DTSSP, the solution was ultrafiltered with CBS buffer (pH 8.5) through an Amicon XM300 membrane. The amination of the reducing group terminal of L-fucose was accomplished through the glycosyl amination reaction. The preparation of liposomes without L-fucose was similar to Fuc-Liposome-Cisplatin except for the process of L-fucose binding.

Physicochemical characterization of Fuc-Liposome-Cisplatin

The average particle size and zeta-potential of liposomes that were prepared in water was determined by dynamic light scattering spectrophotometry (Zetasizer Nano-S90, Malvern, Worcestershire, UK) at 25°C. The instrument was calibrated with standard latex nanoparticles (Malvern, Worcestershire, UK).

Analysis of lipid concentration

Lipid concentrations of Liposome-Cisplatin and Fuc-Liposome-Cisplatin were measured as total cholesterol in the presence of 0.5% Triton X-100 using a Cholesterol E-test Wako kit. The lipid concentration was calculated from the molar ratio of each lipid (4.5) by the following formula (Eq. (1)):

Lipid concentration (mg/mL) = Cholesterol concentration (mg/mL) × 4.5 (1)

Measurement of Cisplatin and calculation of Cisplatin concentration and encapsulation efficiency

Fuc-Liposome-Cisplatin was diluted 10,000-fold with distilled water and the concentration of platinum was measured using an automatic flameless atomic absorption spectrophotometer (FAAS) (Model AA-6700, SHIMADZU, Kyoto, Japan). Potassium dichloroplatinate was used as a standard. A calibration curve with platinum concentrations in the range of 50 - 250 ng/ml was run before analysis of each sample type. The amounts of Cisplatin were calculated by the following formula (Eq. (i)):

Cisplatin concentration = A× (300/195) (i)

where A is the concentration of platinum, 300 is the molecular weight of Cisplatin, and 195 is the molecular weight of platinum. Encapsulation efficiency and the Cisplatin: lipid weight ratio were calculated by the following formula (Eq. (ii)) and (Eq. (iii)) respectively.

Encapsulation efficiency (%) = (Amount of Cisplatin in liposomes/Initial amount of Cisplatin) × 100 (ii)

Cisplatin to lipid weight ratio = Cisplatin concentration (mg/mL)/Lipid concentration (mg/mL) (iii)

*In vitro* cell proliferation assay

Pancreatic cancer cells (2 x 10^4^) were seeded into 24-well plates, cultured for 1 day in RPMI-1640 supplemented with 10% fetal bovine serum, 5% L-Glutamine, and 1% antibiotics. Cells were then incubated with different doses of Fuc-Liposome-Cisplatin. After 2 hours incubation, cells were washed twice with PBS and finally resuspended in RPMI 1640 containing serum and antibiotics. After culture for 72 hours, WST-1 reagent was added and the proliferation assay was performed as previously described^4^. Experiments were repeated in triplicate at least twice.

Effect of Fuc-liposome-Cisplatin on CFU-E/BFU-E colony formation

CD34 cells, which were purchased from Takara Bio Inc, were seeded in MethoCult-H4230 (StemCell Technologies) containing 30% FBS, 1% BSA (bovine serum albumin), 0.9% methylcellulose, 2-mercaptoethanol (10^-4^ M), and 2 mM glutamine. Erythroid stimulation was carried out using 2.5 units/ml human recombinant erythropoietin in a tube containing 4 ml of methylcellulose, before the addition of 100 µl of 44x drug solutions (in 20% FBS-IMDM) and 300 µl of cells (1.5 x 10^6^ /ml). Complete medium (100 µl) was added to the control tubes, whereas the same volume of the vehicle used to prepare the drug dilution was added to the solvent tube at the maximum concentration reached in the final dilutions. Finally, 1 ml of methylcellulose-cell suspension was seeded into 35**-**mm dishes~~,~~ and incubated at 37°C in 5% CO_2_ for 14 days. The final concentrations of drugs were adjusted to 1 µM Cisplatin.

**References**
